# (−)‐Epigallocatechin gallate alleviates chronic unpredictable mild stress‐induced depressive symptoms in mice by regulating the mTOR autophagy pathway and inhibiting NLRP3 inflammasome activation

**DOI:** 10.1002/fsn3.3761

**Published:** 2023-10-30

**Authors:** Yulin Zhang, Hongxian Wu, Chaozhi Xu, Shanqian Li, Yue Hu, Zongyi Zhang, Guixian Wu, Yuling Liu, Lin Yang, Yue Huang, Wenjun Lu, Lina Hu

**Affiliations:** ^1^ School of Public Health Guilin Medical University Guilin China; ^2^ Department of Nutrition Second People's Hospital of Ya'an City Ya'an City Sichuan Province China; ^3^ Department of Cardiology, Zhongshan Hospital Shanghai Institute of Cardiovascular Diseases, Fudan University Shanghai China; ^4^ Medical Information Management, School of Humanities and Management Guilin Medical University Guilin China; ^5^ Guangxi Key Laboratory of Environmental Exposomics and Whole Life Cycle Health Guilin China; ^6^ Key Cultivation Laboratory of Life Cycle Health Care Research Guilin China; ^7^ Institute of Drug Inspection Technology Shanxi Inspection and Testing Center Taiyuan Shanxi Province China; ^8^ Communicable Disease Control Branch Qingdao City Center for Disease Control and Prevention Qingdao China; ^9^ Department of Pediatrics The First Affiliated Hospital of Guangxi Medical University Nanning China; ^10^ General Practice Department Affiliated Hospital of Guilin Medical University Guilin China

**Keywords:** apoptosis, autophagy, chronic unpredictable stress, depression, EGCG, inflammation

## Abstract

Depression is a global public health issue that is widely studied due to the large number of people it affects and its serious consequences. Clinical studies have shown that regular tea consumption may reduce depression risk. (−)‐Epigallocatechin gallate (EGCG), the main tea polyphenol, was observed to alleviate depression, but the underlying mechanism has not been elucidated. In this study, chronic unpredictable mild stress (CUMS) was used to induce depression‐like behavior in mice, and behavioral tests, such as sucrose preference test and forced swim test, were performed. Then, ELISA, western blot and QT‐PCR tests were used to assess the expression of the key components of the NLRP3 inflammasome and its downstream inflammatory effectors (e.g., IL‐1β, IL‐18), autophagy markers (Beclin‐1, LC3, P62) and apoptosis markers (Bax, Bcl‐2) in mouse brain tissues. Changes in serum lipid levels were also assessed. EGCG alleviated CUMS‐induced depression‐like behavioral changes in mice, reduced activation of the NLRP3 inflammasome, inhibited the mTOR signaling pathway, restored autophagy levels, reduced apoptosis marker expression and attenuated abnormal changes in blood lipid levels. Our study demonstrates that EGCG exerts antidepressive effects through multiple mechanisms, providing new insight into the pathological mechanism of depression and laying the foundation for the development of new therapeutic measures.

## Graphical abstract

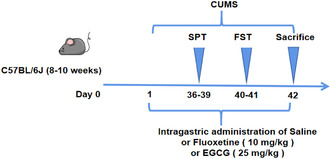

## 1 INTRODUCTION

Major depressive disorder (MDD), referred to as depression, is characterized by strong and lasting symptoms such as low mood, anhedonia, slow thinking, fatigue, a sense of worthlessness or guilt, pessimism and recurrent suicidal thoughts (Riggs et al., 2019). MDD has a high prevalence and recurrence rate. According to the World Health Organization, there are approximately 350 million people suffering from MDD worldwide (Smith, 2014). In the comprehensive ranking of disability and mortality, MDD ranks ninth after heart disease, stroke and AIDS. The years of health “lost” due to disability (years of health “lost” due to physical or mental disability) caused by MDD is 76.4 million years globally, accounting for 10.3% of the total disease burden in the world and representing a serious threat to human life and health (Park & Zarate Jr., 2019). In particular, the COVID‐19 pandemic has increased the burden of mental illness by 27.6% in the prevalence of depression (COVID‐19 Mental Disorders Collaborators, 2021).

At present, the pathogenesis of MDD is not fully understood. It is worth noting that the systemic inflammatory response caused by the activation of the NOD‐like receptor family pyrin domain containing 3 (NLRP3) inflammasome is one of the key pathogenic factors of depression (Wan et al., 2022). The NLRP3 inflammasome is a multiprotein oligomerization composed of the upstream sensor protein NLRP3, the apoptosis‐associated speck‐like protein containing CARD (ASC) adaptor protein and the downstream effector protein Caspase‐1, the latter of which is involved in a variety of host immune and inflammatory responses (Wang & Hauenstein, 2020). Stimulation by chronic unpredictable mild stress (CUMS) activates the NLRP3 inflammasome and caspase‐1, leading to the release of the proinflammatory cytokines interleukin‐18 (IL‐18) and interleukin‐1β (IL‐1β). The NLRP3 inflammasome is involved in the immune activation process, activates the excessive inflammatory response and further leads to MDD (Li, Wang, et al., 2022). However, studies have found that autophagy can sense and eliminate endogenous signals associated with inflammasome activation, which can prevent NLRP3 inflammasome activation and proinflammatory cytokine release, thereby preventing harmful and uncontrolled inflammation and a balanced inflammatory response (Cao et al., 2019).

Autophagy involves a tightly regulated cellular degradation pathway and a stress‐activated pathway that removes damaged organelles and intracellular pathogens to maintain cellular homeostasis (Choi et al., 2013). Under continuous high‐intensity stimulation or excessive inflammation, abnormal expression of the autophagy‐specific proteins, Beclin‐1, microtubule‐associated proteins (MAP1LC3/LC3) and sequestosome‐1 (SQSTM1/P62), impairs the autophagy pathway and further promotes the occurrence of depression (Tang et al., 2021). In addition, Yang et al. (2019) reported that inhibition of the mammalian target of rapamycin (mTOR) signaling pathway in diabetic mice improves autophagy and inhibits the activation of the NLRP3 inflammasome, thereby exerting cardiac protection. Moreover, some studies (Cosin‐Roger et al., 2017) have found that hypoxia can improve intestinal inflammation by downregulating the expression of mTOR and NLRP3 and by activating autophagy.

mTOR, a serine/threonine protein kinase, is the main regulator of pathways involved in cellular metabolism and autophagy and is the main kinase that inhibits autophagy (Kim & Guan, 2015). In fact, mTOR dysregulation is closely related to diseases involving autophagy defects. For example, in depression, the antidepressant fluoxetine can effectively reverse depression‐like behavioral changes in animal models by inhibiting mTOR activation (Zhou et al., 2019), suggesting that autophagy activation is conducive to reversing depression.

At the same time, dysfunctional autophagy can activate the apoptotic signaling pathway and promote cell death, leading to severe neurobehavioral changes in mice (Kwatra et al., 2021). In addition, some studies have reported that dyslipidemia is closely related to depression, leading to the aggravation of suicidal tendencies in patients with depression (Lee et al., 2022). Fluoxetine can correct abnormal lipid metabolism and enhance autophagy in the hippocampus, thus reversing depression‐like behavior after olfactory bulb resection in rat models of depression (Zhou et al., 2019). Therefore, we speculate that pleiotropic antidepressant drugs that reduce mTOR signaling, promote autophagy, inhibit NLRP3 inflammasome activation and excessive inflammatory responses, reduce apoptosis and improve dyslipidemia will be beneficial for treating depression.

Antidepressant drugs commonly used in clinical practice are associated with many side effects, such as addiction, headache and sexual dysfunction, and are associated with unsatisfactory efficacy (Liu et al., 2019; Tang & Li, 2020). Therefore, it is necessary to find safe pleiotropic antidepressant drugs without adverse metabolic effects. With consideration given to tolerability and safety, studies aimed at the discovery and development of antidepressant drugs and nonpharmacological treatment measures (e.g., psychological intervention, physical exercise, improved diet) in Chinese herbal medicine are being pursued by researchers (Limanaqi et al., 2020; Yeung et al., 2018). Among these interventions, diet is the most cost‐effective and efficacious. An increasing number of studies have found that drinking tea has many benefits, including antiaging and antidiabetic effects that promote metabolism and cardiovascular health (Bag et al., 2022). Moreover, international dietary guidelines recommend that moderate daily tea intake contributes to physical and mental health. Studies have confirmed that regular tea drinking is associated with antidepressant effects, possibly related to polyphenols in tea, which act simultaneously through a variety of mechanisms to reduce the risk of MDD (Rothenberg & Zhang, 2019). (−)‐Epigallocatechin gallate (EGCG, C_22_H_18_O_11_) is the most abundant catechin in green tea (Saeki et al., 2018) and is regarded as a potent antioxidant. It has been widely used in the prevention and treatment of various diseases because of its multiple effects, such as anti‐inflammation, anti‐apoptosis and autophagy promotion (Chakrawarti et al., 2016). However, the underlying mechanisms of EGCG's antidepressant effects have not been fully elucidated. Therefore, in this study, the chronic unpredictable mild stress (CUMS) model (Hao et al., 2019) was used to explore the mechanism of action associated with the antidepressant effect of EGCG and its effect on blood lipid levels. We hypothesized that EGCG alleviates CUMS‐induced depressive symptoms in mice by regulating the mTOR autophagy pathway, inhibiting NLRP3 inflammasome activation, reducing apoptosis and improving dyslipidemia.

## 2 MATERIALS AND METHODS

### 2.1 Animals

Twenty‐four specific pathogen‐free (SPF) male C57BL/6J mice, aged 8–10 weeks and weighing 18–25 g, were purchased from Hunan Anshengmei Pharmaceutical Research Institute Co., Ltd. (license number: SCXK Xiang: 2019‐0004). Before the start of the experiment, the mice were placed in the SPF animal room of the Guangxi Key Laboratory of Environmental Exposomics and Life‐Cycle Health at Guilin Medical College. The mice were adaptively fed for 1 week (room temperature: 20–24°C, humidity: 40%–60%, circadian cycle: 12 h). Approval for this experiment was obtained from the animal ethics review board at Guilin Medical University on March 15, 2022 (audit no. GYLL2022050). All experiments complied with the ethical requirements of animal experimentation and relevant administrative regulations.

### 2.2 Drug treatment

All mice were randomly divided into 4 groups (*n* = 6, each) as follows: control group, saline group (saline+CUMS), fluoxetine group (10 mg/kg/day fluoxetine+CUMS) and EGCG group (25 mg/kg/day EGCG+CUMS). Fluoxetine and EGCG were obtained from Sigma–Aldrich Chemical Co. One week before the CUMS procedure, mice were given drugs by gavage corresponding to treatment assignment 1 h prior to beginning the CUMS procedure (between 8:00 and 9:00) every morning for 6 weeks. Body weight was monitored daily throughout the whole process.

### 2.3 CUMS procedure

**FIGURE 1:**
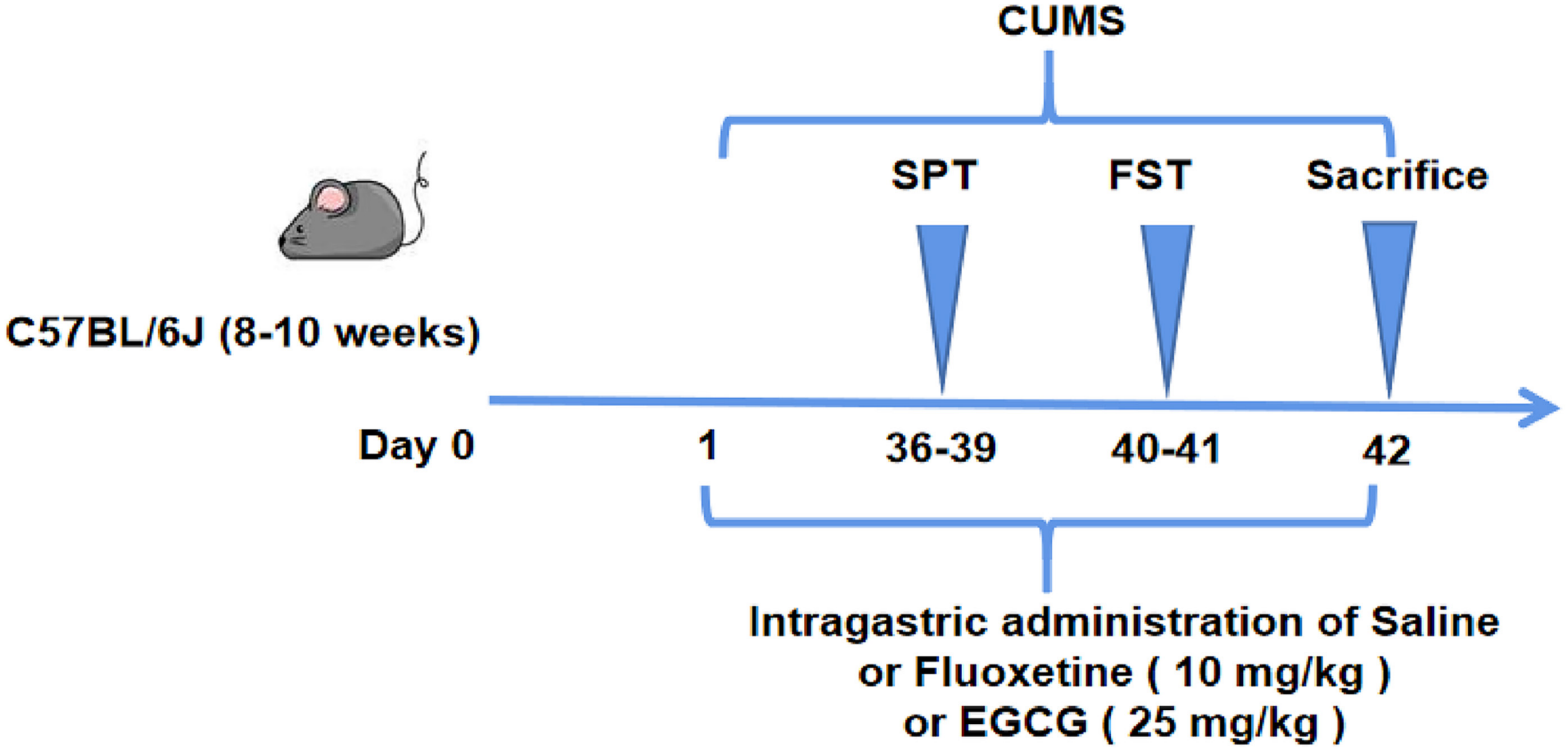
Study design, drug schedule. FST was followed immediately after the SPT. CUMS, chronic unpredictable mild stress; FST, forced swim test; SPT, sucrose preference test.

To avoid circadian rhythm effects, mice in all groups except the control group were randomly subjected to two different stimuli at a fixed time (from 9 a.m. to 13 p.m.) daily after the one‐week adaptation period to prepare for the CUMS depression model (Hao et al., 2019). Specific stressors included restraint in a 50 mL centrifuge tube for 4 h, social stress (crowding for 24 h or isolation for 24 h), tail clamping (10 min), rapid light and dark change, overnight light exposure, cage tilt 45° for 6 h, day/night reversal, wet bedding for 6 h (new bedding 100 g plus 200 mL water) and removal of bedding. CUMS was continuously present for 6 weeks (Table 1). All experimental plans are shown in Figure 1.

**TABLE 1:** Chronic unpredictable mild stress(CUMS)procedure.

Stressor	Duration
Physical restraint	4 h
Bedding removal	6 h
Social stress (crowding/isolated housing)	24 h
Tail pinch	10 min
45 cage tilt	6 h
Wet cage	6 h
Illumination overnight	12 h
Reversal of day and night	24 h

### 2.4 Assessments of depression‐like behaviors

#### 2.4.1 Sucrose preference test (SPT)

The sucrose preference test (SPT) was performed according to previous reports (He et al., 2020). In brief, mice were housed individually and were adaptively fed via two bottles of 1% sucrose water per cage for 24 h. Then, one bottle was replaced with pure water for 24 h, and the bottle positions were switched every 12 h. After 24 h of water deprivation, the mice were then exposed to a bottle of 1% sucrose water and a bottle of pure water for 24 h, with the bottle position changing every 12 h. Within 24 h of testing, the levels of pure water and sucrose water consumption were measured, and the sucrose preference rate was calculated, with SPT (%) = sucrose intake (g)/[water intake (g) + sucrose intake (g)]*100%.

#### 2.4.2 Forced swim test (FST)

The forced swim test (FST) was performed according to a previously reported protocol (Bogdanova et al., 2013). In short, mice were placed into a plexiglass circle (20 cm high, 14 cm diameter) for swimming. The cylinder contained 16 cm high fresh water (25°C), and the room temperature was maintained at approximately 23°C. The total time of each forced swim test was 6 min, with 2 min of adaptation to swimming and 4 min of immobility time. Immobility time was defined as the time when the mouse floated motionless on the water surface with only a slight swing of its tail and forepaws to maintain body balance and keep its head above water.

### 2.5 ELISA was used to detect NLRP3 in the brain

Mice were anesthetized and dissected by intraperitoneal injection of sodium pentobarbital (50 mg/kg), and the whole mouse brain was extracted for the preparation of brain tissue homogenate. The level of NLRP3 in brain tissue homogenate was determined using a standard sandwich enzyme‐linked immunosorbent assay (ELISA) kit (Huamei Bioengineering Co., Ltd.) according to the manufacturer's instructions. The results were evaluated with a standard curve and are presented graphically as pg/mL.

### 2.6 Western blot was used to assess the expression of related proteins in brain tissue

The expression levels of autophagy‐, apoptosis‐ and inflammation‐related proteins in brain tissue were assessed with western blotting (WB). Brain tissue protein supernatants were extracted using lysates containing protease inhibitors. A BCA kit (Shanghai Biyuntian Institute of Biotechnology, Shanghai, China) was used to determine the concentration of protein in the supernatant. Proteins were then isolated and separated by sodium dodecyl sulfate–polyacrylamide gel electrophoresis (SDS–PAGE). Proteins were subsequently transferred to PVDF membranes (Merck Millipore Inc.) and incubated with goat anti‐B‐cell lymphoma‐2 (Bcl‐2), Bcl‐2‐associated X (Bax), mTOR, Beclin‐1, p62, LC3, ASC, Caspase‐1, IL‐1β and NLRP3 primary antibodies (CST, 1:1000) overnight at 4°C. The membranes were then incubated with horseradish peroxidase‐conjugated secondary antibodies at a dilution of 1:10,000 to15,000 for 1 h at room temperature. Finally, the level of target protein was detected by a fully automated gel imaging system (Protein Simple Inc.). Protein levels quantified by western blotting were normalized to the internal control glyceraldehyde 3‐phosphate dehydrogenase (GAPDH; CST, 1:1000).

### 2.7 Quantitative real‐time polymerase chain reaction (QT‐PCR) was used to assess the mRNA expression levels of related factors in brain tissue

The mRNA expression levels of inflammatory‐, autophagy‐ and apoptosis‐related factors were evaluated by QT‐PCR. Total RNA was extracted from brain tissue with a TRIzol kit (Invitrogen), and cDNA was synthesized by reverse transcription using a third‐generation reverse transcription premix (without primers; Monad Biotech Co., Ltd.) according to the manufacturer's instructions. Quantitative PCR premix (high ROX; Monad Biotech Co., Ltd.) was used for real‐time PCR analysis. Data were normalized according to the expression of GAPDH in the corresponding samples. The thermocycling program consisted of 40 cycles for 10 s at 95°C, 10 s at 60°C and 30 s at 72°C. A summary of the targeted genes and primers is shown in Table 2.

**TABLE 2:** Summary of targeted genes and primers.

Primer	Forward primer (5′– > 3′)	Reverse primer (5′– > 3′)
mTOR	CAGTTCGCCAGTGGACTGAAG	GCTGGTCATAGAAGCGAGTAGAC
Beclin‐1	ATGGAGGGGTCTAAGGCGTC	TGGGCTGTGGTAAGTAATGGA
LC3a	GACCGCTGTAAGGAGGTGC	CTTGACCAACTCGCTCATGTTA
LC3b	TTATAGAGCGATACAAGGGGGAG	CGCCGTCTGATTATCTTGATGAG
P62	GAACTCGCTATAAGTGCAGTGT	AGAGAAGCTATCAGAGAGGTGG
Bax	AGACAGGGGCCTTTTTGCTAC	AATTCGCCGGAGACACTCG
Bcl‐2	GCTACCGTCGTGACTTCGC	CCCCACCGAACTCAAAGAAGG
NLRP3	ATCAACAGGCGAGACCTCTG	GTCCTCCTGGCATACCATAGA
IL‐1β	GAAATGCCACCTTTTGACAGTG	TGGATGCTCTCATCAGGACAG
IL‐18	GACTCTTGCGTCAACTTCAAGG	CAGGCTGTCTTTTGTCAACGA
ASC	GACAGTGCAACTGCGAGAAG	CGACTCCAGATAGTAGCTGACAA
TLR‐4	ATGGCATGGCTTACACCACC	GAGGCCAATTTTGTCTCCACA
TLR‐2	TCTAAAGTCGATCCGCGACAT	CTACGGGCAGTGGTGAAAACT
TNF‐α	CTGAACTTCGGGGTGATCGG	GGCTTGTCACTCGAATTTTGAGA
CXCR‐4	GACTGGCATAGTCGGCAATG	AGAAGGGGAGTGTGACAAA
GAPDH	AAGAAGGTGGTGAAGCAGG	GAAGGTGGAAGAGTGGGAGT

### 2.8 Lipid content

Serum levels of total cholesterol (TC), triglycerides (TC), low‐density lipoprotein cholesterol (LDL‐C) and high‐density lipoprotein cholesterol (HDL‐C) were evaluated using a kit (Ilarite Biotechnology Co., Ltd.).

### 2.9 Statistical analysis

GraphPad Prism 8 statistical software was used for data analysis, and the data are expressed as the mean ± SEM. The t test and one‐way ANOVA were used to assess differences between groups. Tukey's test was used to evaluate multiple comparisons, and *p* < .05 was considered statistically significant.

## 3 RESULTS

### 3.1 EGCG mitigates depression‐like behavior induced by CUMS in mice

**FIGURE 2:**
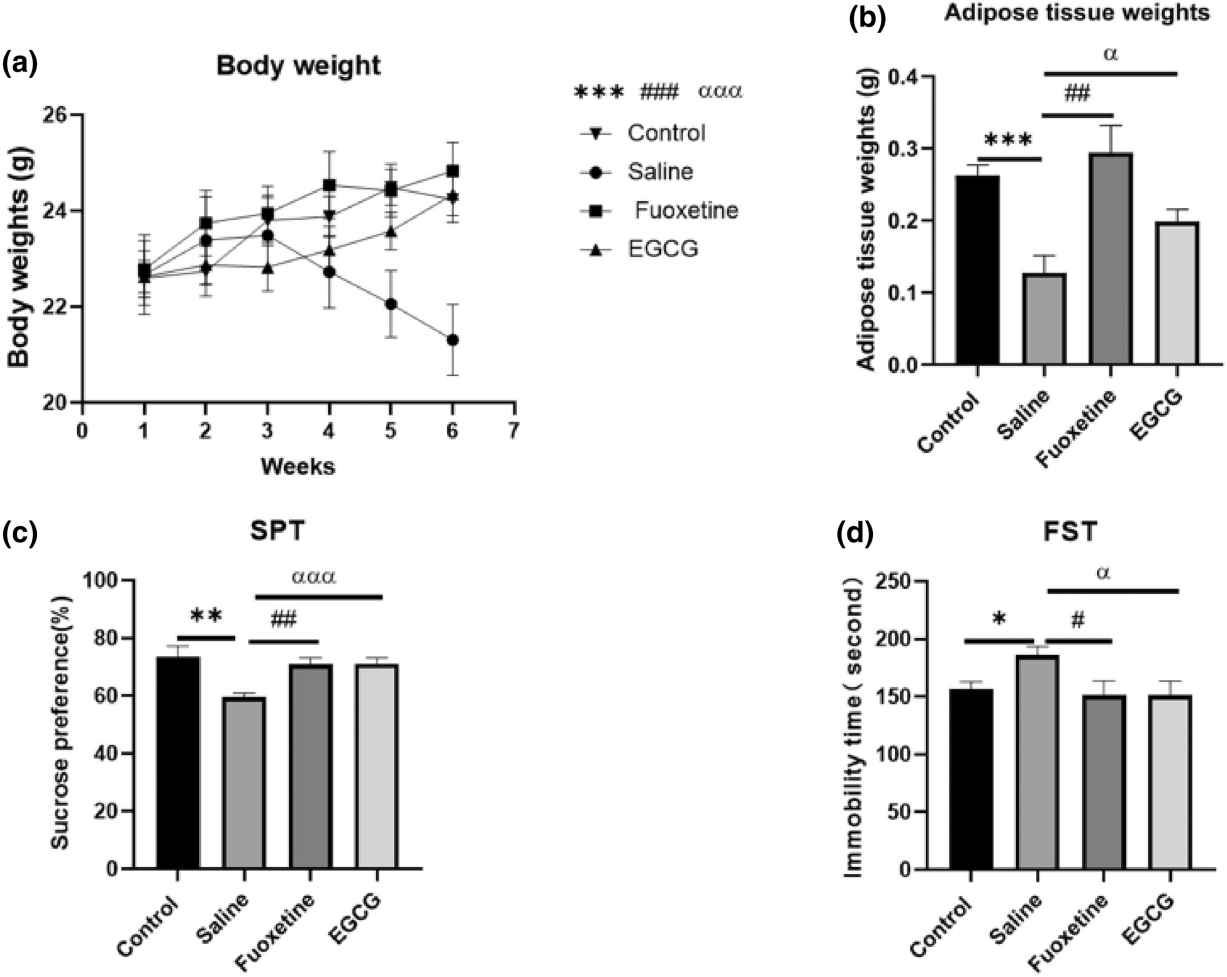
(a) Relative body weight analysis, (b) epididymal visceral adipose tissue weight, (c) sucrose preference test and (d) forced swim test. (*): CUMS compared with control, (#): fluoxetine compared with saline‐treated mouse, (α): EGCG compared with saline‐treated mouse. *p* < .05 was considered statistically significant. CUMS, chronic unpredictable mild stress; FST, forced swim test; SPT, sucrose preference test. (*): *p* < .05, (**): *p* < .01, (***): *p* < .001; (#): *p* < .05, (##): *p* < .01, (###): *p* < .001; (α): *p* < .05, (αα): *p* < .01, (ααα): *p* < .001.

CUMS is a classical model for screening antidepressants and studying the pathophysiology of depression (Hao et al., 2019). Adult C57BL/6J mice treated with CUMS can induce a variety of behavioral and physiological depression‐like symptoms. Therefore, in this study, CUMS‐induced depression‐like behavior in mice was evaluated by measuring body weight (Figure 2a), visceral fat content of the epididymis (Figure 2b), SPT (Figure 2c) and immovable time as assessed by the FST (Figure 2d). CUMS‐treated mice had decreased body weight (*p* < .001) and epididymal visceral fat content (*p* < .001). However, this change was attenuated after intervention with EGCG (25 mg/kg) or fluoxetine (10 mg/kg; Figure 2a,b). The results of the SPT and the FST have shown that CUMS treatment significantly decreased the sucrose preference rate (*p* < .01) and increased the immobility time (*p* < .05). This was significantly reversed with EGCG or fluoxetine treatment (Figure 2c,d). In summary, EGCG significantly improves anhedonia and behavioral despair induced by CUMS in mice.

### 3.2 EGCG alleviates CUMS‐induced hyperinflammation in mouse brain tissue

**FIGURE 3:**
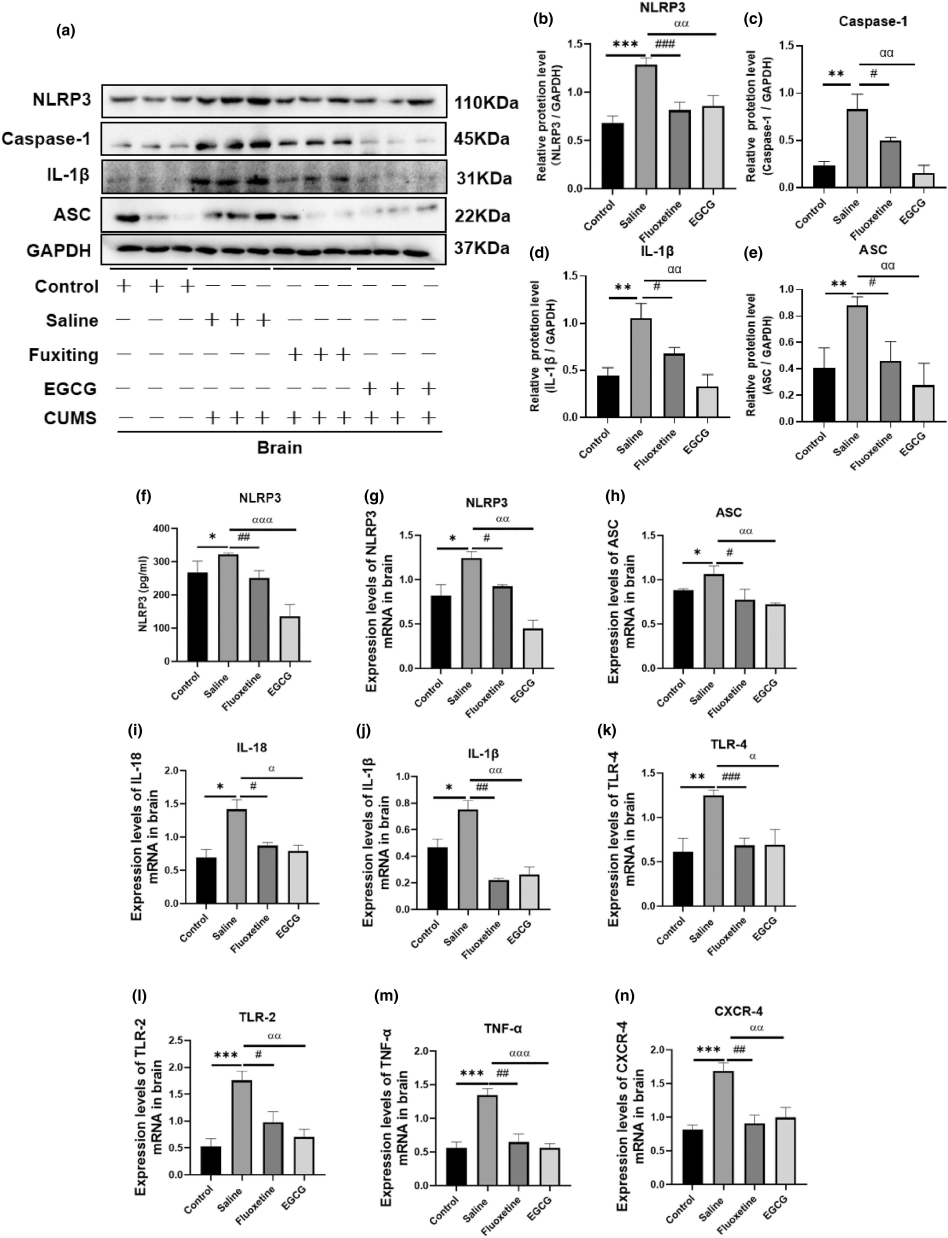
EGCG reverses the CUMS‐mediated upregulation of NLRP3 and its downstream proinflammatory factors. (a) The representative images of western blot showed the intensities of NLRP3, Caspase‐1, IL‐1β and ASC bands in mice brain tissue (*n* = 3). (b–e) ImageJ software quantitated protein imprinting of NLRP3, Caspase‐1, IL‐1β, ASC in mice brain tissue (*n* = 3). (f) The expression activity of NLRP3 in the mice brain was detected by ELISA (*n* = 3). (g–n) Relative mRNA expression of NLRP3, ASC, IL‐18, IL‐1β, TLR‐4, TLR‐2, TNF‐α and CXCR‐4 in brain tissue was determined by QT‐PCR (*n* = 3). The data are expressed as the mean ± SD, and the results were analyzed by one‐way ANOVA, followed by post hoc analysis. (*): CUMS compared with the control group, (#): fluoxetine compared with the saline group, (α): EGCG compared with the saline group. *p* < .05 was considered statistically significant. (*) *p* < .05, (**): *p* < .01, (***): *p* < .001; (#): *p* < .05, (##): *p* < .01, (###): *p* < .001; (α): *p* < .05, (αα): *p* < .01, (ααα): *p* < .001.

Exposure to psychological or physiological stressors can activate inflammatory responses, and CUMS activates the NLRP3 inflammasome, stimulating the production of proinflammatory factors, including IL‐1β (Feng et al., 2019). As shown in Figure 3, the results of WB, ELISA and PCR concurrently showed that the protein activity and mRNA expression of NLRP3 (*p* < .05) were increased in the brain tissue of CUMS mice compared to the brain tissues of control mice. WB results also showed that CUMS caused increased protein levels of ASC (*p* < .05), Caspase‐1 (*p* < .05) and IL‐1β (*p* < .05) in mice brain tissue. Meanwhile, PCR results showed that CUMS increased mRNA expression levels of other proinflammatory factors in brain tissue, such as ASC (*p* < .05), interleukin‐18 (IL‐18; *p* < .05), IL‐1β (*p* < .05), toll‐like receptor 4 (TLR‐4; *p* < .01), TLR‐2 (*p* < .001), tumor necrosis factor α (TNF‐α; *p* < .001) and recombinant chemokine C‐X‐C motif chemokine receptor 4 (CXCR‐4; *p* < .05). However, treatment with EGCG or fluoxetine significantly reduced the expression of proinflammatory factors. This suggests that EGCG and fluoxetine significantly inhibit NLRP3 activation in a stress‐induced mouse depression model, which hinders the inflammatory response and reduces depressive symptoms.

### 3.3 EGCG improves CUMS‐induced autophagy injury issues in the mouse brain

**FIGURE 4:**
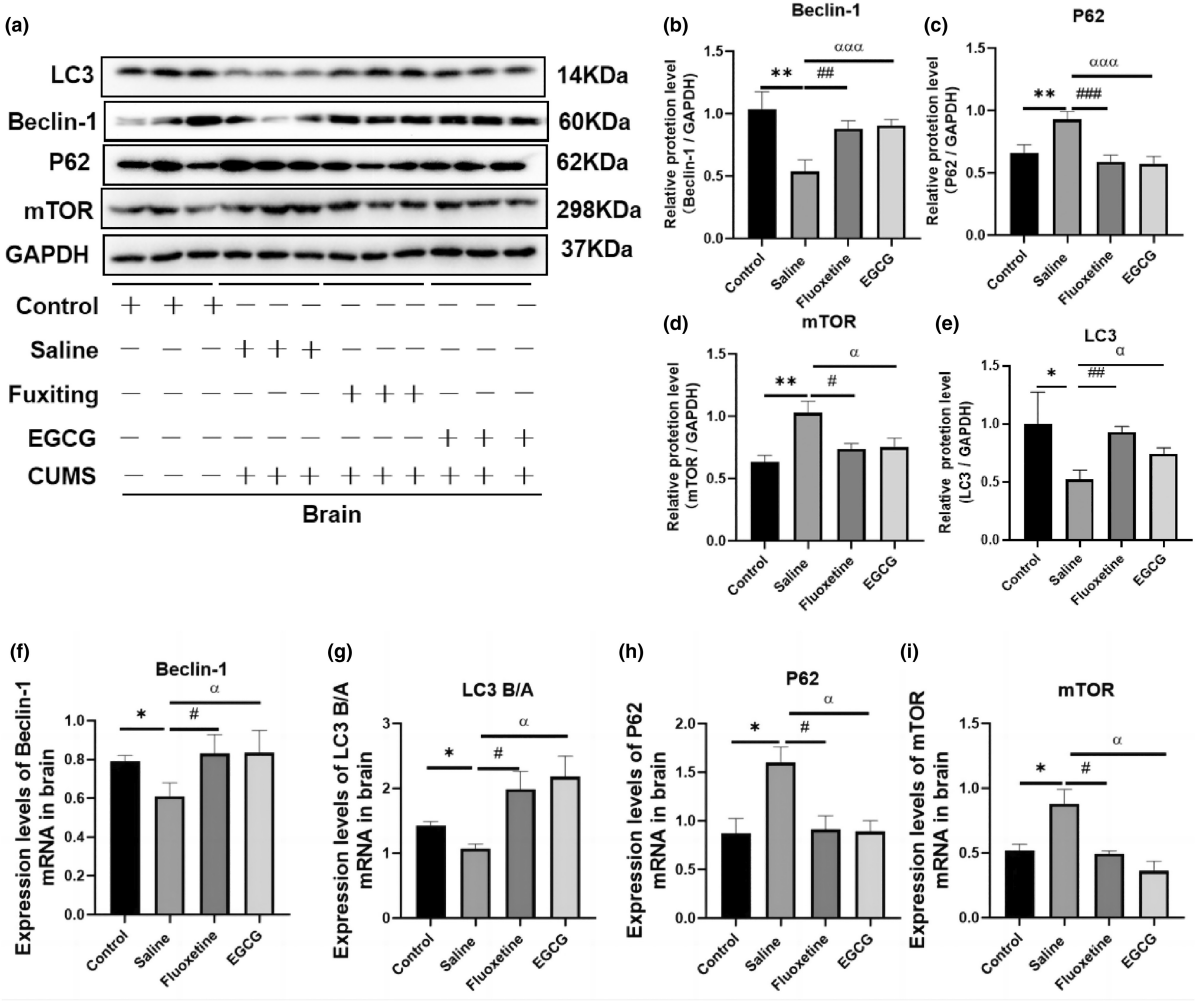
EGCG reversed CUMS‐induced changes in autophagy‐related factor expression. (a) The representative images of western blots showed the intensities of mTOR, Beclin‐1, P62 and LC3 bands in mice brain tissue (*n* = 3). (b–e) ImageJ software quantitated protein imprinting of mTOR, Beclin‐1, P62, LC3 in mice brain tissue (*n* = 3). (f–i) Relative mRNA expression of Beclin‐1, LC3B/A, P62 and mTOR in brain tissue was determined by QT‐PCR. The data are expressed as the mean ± SD, and the results were analyzed by one‐way ANOVA, followed by post hoc analysis. (*): CUMS compared with the control group, (#): fluoxetine compared with the saline group, (α): EGCG compared with the saline group. *p* < .05 was considered statistically significant. (*) *p* < .05, (**) *p* < .01; (#) *p* < .05, (##) *p* < .01, (###) *p* < .001; (α) *p* < .05, (ααα) *p* < .001.

Autophagy is a process involving biological renewal and metabolism. Chronic stress induces autophagic injury in the body, which promotes the occurrence of depression (Li, Wang, et al., 2022). Therefore, we evaluated the expression of autophagy‐related factors during CUMS‐induced exposure. The WB and PCR results showed that CUMS exposure altered the expression of autophagy‐related signaling molecules, including significant reduced the levels of Beclin‐1 (*p* < .05) and LC3 mRNAs and proteins (*p* < .05), and remarkably increased the levels of P62 (*p* < .05) and mTOR mRNAs or/and proteins (*p* < .001). Nevertheless treatment with EGCG or fluoxetine resulted in a significant reversal of these changes (Figure 4a–i). These data suggest that CUMS treatment causes dysregulated autophagy in mouse brain tissue and that EGCG treatment can restore autophagy by inhibiting the mTOR pathway.

### 3.4 EGCG reduces CUMS‐induced apoptosis in mouse brain tissue

**FIGURE 5:**
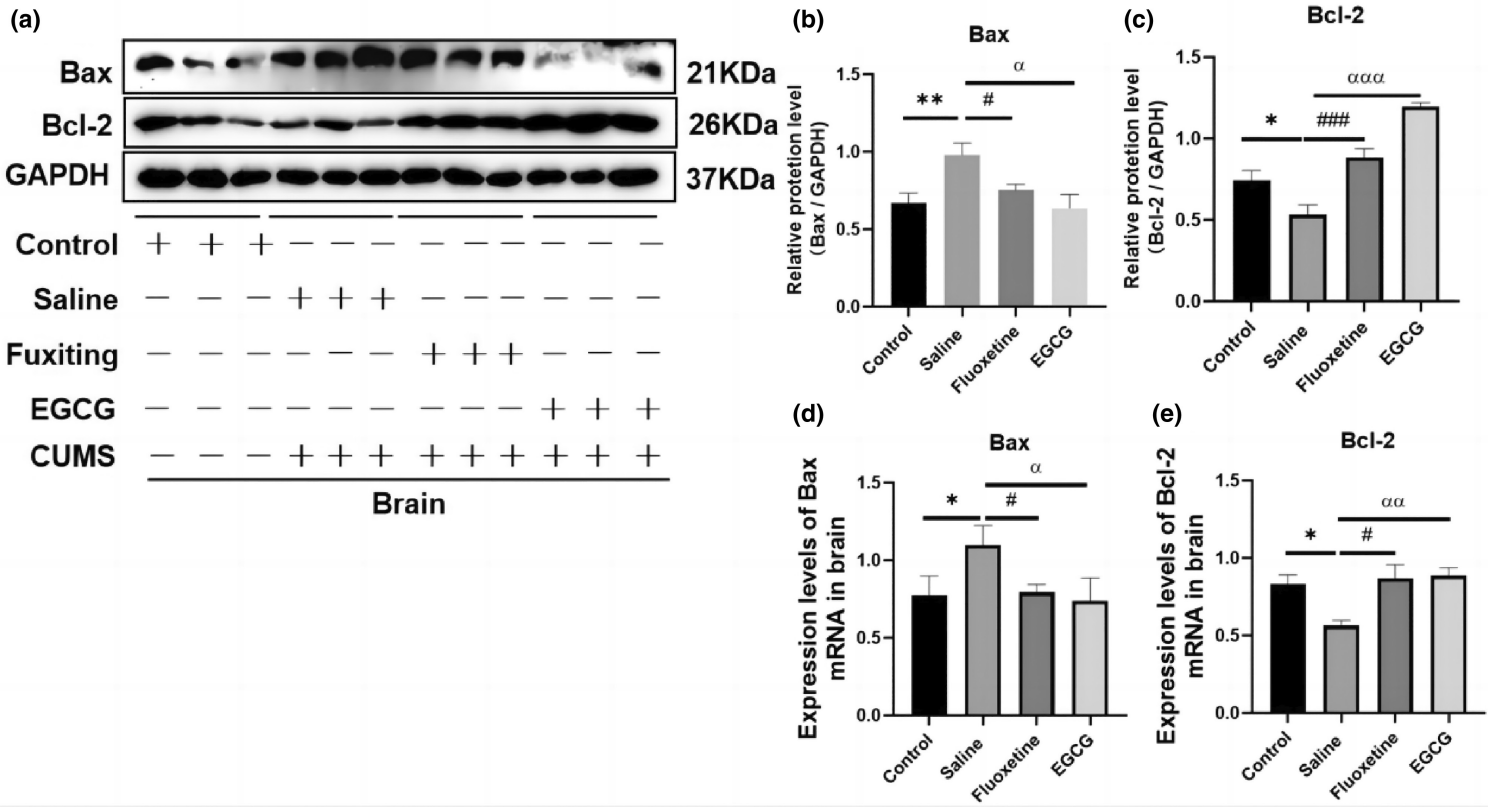
EGCG treatment eliminated the effect of CUMS on apoptosis‐related factors. (a) Western blotting showing the protein levels of Bax and Bcl‐2 in brain tissue (*n* = 3); (b,c) histogram quantifying the protein levels of Bax and Bcl‐2 in brain tissue (*n* = 3); (d,e) relative mRNA expression of Bax and Bcl‐2 in brain tissue (*n* = 3). Western blotting was used to analyze protein expression, ImageJ software was used for quantitative imprinting and QT‐PCR was used to measure the expression of related mRNAs. The data are expressed as the mean ± SD, and the results were analyzed by one‐way ANOVA, followed by post hoc analysis. (*): CUMS compared with the control group, (#): fluoxetine compared with the saline group, (α): EGCG compared with the saline group. *p* < .05 was considered statistically significant. (*) *p* < .05, (**): *p* < .01; (#): *p* < .05, (###): *p* < .001; (α): *p* < .05, (αα): *p* < .01, (ααα): *p* < .001.

The research finding suggests increased apoptosis and different regulation of pro‐apoptosis protein Bax and anti‐apoptosis protein Bcl‐2 in the olfactory bulb of a rat model of depression (Yang et al., 2011). Therefore, we examined Bax and Bcl‐2 levels in mouse brain tissue to assess apoptosis. We found that CUMS increased the expression of Bax protein (*p* < .05) and significantly decreased the expression of Bcl‐2 protein (*p* < .05). EGCG or fluoxetine treatment resulted in significant recovery of the changes observed in Bax (*p* < .01) and Bcl‐2 (*p* < .001; Figure 5a–c). In addition, verification of these results at the mRNA level was confirmed by QT‐PCR (Figure 5d,e). These results suggest that CUMS induces brain hyperapoptosis in mice and that EGCG may improve depression‐like behavior by inhibiting this deleterious effect.

### 3.5 EGCG alleviates the effect of CUMS on serum lipids in mice

**FIGURE 6:**
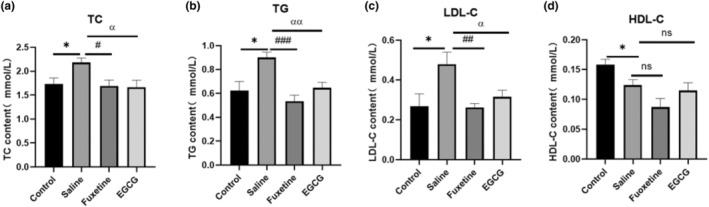
EGCG reversed CUMS‐mediated serum dyslipidemia in mice. (a–d) Serum total cholesterol (TC), triglyceride (TG), low‐density lipoprotein cholesterol (LDL‐C) and high‐density lipoprotein cholesterol (HDL‐C) levels in mice (*n* = 6). The data are expressed as the mean ± standard deviation, and the results were analyzed by one‐way ANOVA, followed by post hoc analysis. *p* < .05 was considered statistically significant. (*): CUMS compared with the control group, (#): fluoxetine compared with the saline group, (α): EGCG compared with the saline group. *p* < .05 was considered statistically significant.(*): *p* < .05; (#): *p* < .05, (##): *p* < .01, (###): *p* < .001; (α): *p* < .05, (αα): *p* < .01, (ns): *p* > .05.

At present, although consensus is lacking regarding the relationship between depression and changes in serum lipids, many studies have indicated that increased depressive symptoms are associated with elevations in total cholesterol (TC), triglycerides (TG) and low‐density lipoprotein cholesterol (LDL‐C) and with low high‐density lipoprotein cholesterol (HDL‐C) (Lee et al., 2022; Ye et al., 2022). To assess the relationship between depression and blood lipids and the effect of EGCG on blood lipids, serum lipid levels in CUMS‐induced depression mice were examined using an enzyme colorimetric kit. The results shown CUMS‐induced increases in serum TC (*p* < .05), TG (*p* < .05) and LDL‐C (*p* < .05; Figure 6a–c) and improvement in these abnormal changes in blood lipids with EGCG or fluoxetine treatment, but the level of HDL‐C in serum was not statistically significant (*p* > .05; Figure 6d).

## 4 DISCUSSION

The CUMS animal depression model is a classic model for the screening of antidepressant drugs and evaluation of the depression of pathophysiology (Hao et al., 2019). The model mimics many physical and mental conditions of the human body under chronic social and psychological stress in modern life, and it can present occurrence and development of depression. Our results clearly showed that CUMS mice exhibited typical depression‐like behaviors such as weight loss, anhedonia (decreased sucrose preference rate in SPT) and learned helplessness (increased immobility time during the FST).

Autophagy has been shown to lower chronic inflammation, which ameliorates the development of depression (Cao et al., 2019; Li, Wang, et al., 2022; Tang et al., 2021). It was reported that 25 mg/kg of EGCG can protect cystic fibrosis by the activation of autophagy (Caution et al., 2019). Other studies have demonstrated that 20 mg/kg of EGCG effectively activates autophagy, reduced visceral fat accumulation in mice induced by diet (Choi et al., 2020). Considering the close association between autophagy deficits, dyslipidemia and depression, we hypothesized that EGCG at 25 mg/kg may improve lipid levels by the activation of autophagy, leading to alleviation of CUMS‐induced depression in mice. Therefore, in this study, we used the dose of 25 mg/kg of EGCG on CUMS mice.

Our results show that continuous CUMS stimulation induced the activation of NLRP 3 inflammatory corpuscles in brain tissue, and the expression of ASC, Caspase‐1, IL‐1β and IL‐18 protein and mRNA was significantly increased. Similarly, previous studies have shown that chronic stress mediates systemic inflammatory responses in mice by activating NLRP 3 inflammatory corpuscles and releasing the pro‐inflammatory factors IL‐18 and IL‐1β, thereby further inducing depression‐like behavioral changes in mice (Feng et al., 2019). This is consistent with our findings. In addition, some studies have reported that increased TNF‐α expression selectively affects the anatomical changes of brain structures in patients with depression (Zhou et al., 2018). One study found (Ogłodek et al., 2014) an increase in CXCR‐4 receptors in patients with different types of depression. Keri et al. found that the TLR‐4 signaling pathway was up‐regulated in patients with depression and down‐regulated after cognitive behavioral therapy, but TLR‐2 expression was not changed (Kéri et al., 2014). In this study, we also observed the mRNA levels of TNF‐α, TLR‐2, TLR‐4 and CXCR‐4 were all significantly increased in mice brain tissues. In addition, we found that the administration of EGCG significantly reversed CUMS‐induced activation of NLRP3 inflammasome and proinflammatory factors. Similarly Arioz et al. showed that inhibiting activation of the NLRP3 inflammasome, preventing ASC formation, caspase‐1 cleavage and IL‐1β maturation and secretion can alleviate acute depressive‐like behavior in mice (Arioz et al., 2019), which is in agreement with our results. The analysis indicates that the antidepressant effect of EGCG is at least partially mediated by inhibiting NLRP3 inflammasome activation and reducing the inflammatory response.

Besides, autophagy plays an important role in the occurrence and development of depression. Wang et al. (2020). found that promoting autophagy by restraining the adenosine 5′‐monophosphate (AMP)‐activated protein kinase (AMPK) pathway and attenuated NLRP3 inflammasome activation, leading to the neuroprotective and antidepressant effects. Moreover, many studies have shown that the damage of the autophagy degradation pathway plays an important role in the inflammatory response activated by the NLRP3 inflammasome. Autophagy‐related proteins such as P62 and Beclin‐1 can directly interact with inflammatory pathways during the interaction between autophagy and inflammation (Biasizzo & Kopitar‐Jerala, 2020). Meanwhile, Jia et al. (2022) found that EGCG attenuated myocardial fibrosis in diabetic rats, through regulating the AMPK/mTOR pathway, upregulating the expression of LC3 and Beclin‐1 and activating autophagy. Therefore, we hypothesized that the antidepressant effect of EGCG might be related to the activation of the autophagy pathway and the reduction in NLRP3 inflammasome activation. Here we found that continuous CUMS exposure activated the mTOR pathway, which greatly increased the protein and mRNA levels of autophagy inhibitor P62, significantly decreased the expression of autophagy promoters LC3 and Beclin‐1 and caused autophagy impairment. Nevertheless, EGCG intervention inhibited the mTOR signaling pathway, reversed these changes and restored autophagy.

Autophagy and apoptosis interact to regulate cell death and survival during adaptation and response to stress. Autophagy can prevent tissue inflammation by eliminating apoptotic bodies. As demonstrated by studies by Rong et al. (2019), neural stem cell‐derived small extracellular vesicles attenuate apoptosis and neuroinflammation after traumatic spinal cord injury by activating autophagy. Similarly, evidence points to the upregulation of Bax protein expression and the downregulation of Bcl‐2 protein observed in a CUMS depression rat model, while Bax downregulation reduces anxiety and depression‐like behaviors in a rat model of chronic stress (Yang et al., 2011), which is consistent with our findings. Our results have shown that CUMS exposure significantly increased the expression of the proapoptotic factor Bax and decreased the expression of the antiapoptotic factor Bcl‐2. Several studies have shown that EGCG exhibits antiapoptotic properties in different cell types (Jia et al., 2020; Liu et al., 2017). Likewise, in this study, EGCG intervention markedly restored the CUMS‐induced changes described above, inhibiting excessive apoptosis in brain tissue. These data suggest that EGCG alleviates depression‐like behaviors at least in part by inhibiting CUMS‐induced excessive apoptosis in brain tissues.

In addition, clinical studies have confirmed that abnormal changes in blood lipid levels are closely related to the occurrence of depression. Recent clinical observations showed that the harmful changes in the plasma TG, TC, LDL, glucose and HDL were associated with suicidal tendency in patients with depression (Lee et al., 2022; Ye et al., 2022). Meanwhile, the dysregulation of lipid metabolism is observed in patients with depression. It was reported that the inhibition of autophagy by AMPK/mTOR pathway inactivation leads to depression and anxiety‐like behaviors in high‐fat diet‐induced obese mice (Li, Cheng, et al., 2022). It has been suggested that enhancing autophagy to improve lipid metabolism through the AMPK/mTOR pathway may be a potential therapeutic target for the treatment of depression in obese patients (Khedr et al., 2022). In addition, the research of Huang et al. (2022). proposes that EGCG delayed the progression of coronary heart disease by improving serum lipids in mice, which is consistent with our findings. Our study has shown that CUMS induced an increase in the levels of serum TC, TG and LDL‐C in mice except HDL‐C, while treatment with EGCG reversed CUMS‐induced dyslipidemia. In short, EGCG may also promote autophagy through the mTOR signaling pathway to improve lipid levels and alleviate depressive symptoms.

## 5 CONCLUSION

Our study has shown that EGCG inhibits CUMS‐induced NLRP3 inflammasome activation and excessive apoptosis mediated in mouse brain tissue by inhibiting the mTOR signaling pathway and promoting autophagy while mitigating abnormal changes in blood lipid levels and alleviating depression‐like symptoms in mice. These data suggest that EGCG can alleviate symptoms in patients with depression by regulating multiple pathways through promoting autophagy, thus promoting health. In addition, compared with standard antidepressant drugs, the EGCG used in the current study exerted antidepressant effects without toxic side effects and, therefore, may be more suitable for use in various populations. In recent years, healthy dietary strategies, such as the DASH diet, have suggested that adding light tea intake to the diet can promote health. Future studies on EGCG should focus on the dosage that is appropriate for the human body and on improving EGCG bioavailability.

## AUTHOR CONTRIBUTIONS

**Yulin Zhang:** Conceptualization (equal); data curation (equal); formal analysis (equal); investigation (equal); methodology (equal); visualization (equal); writing – original draft (equal); writing – review and editing (equal). **Hongxian Wu:** Conceptualization (equal); data curation (equal); formal analysis (equal); investigation (equal); methodology (equal); visualization (equal); writing – original draft (equal); writing – review and editing (equal). **Chaozhi Xu:** Data curation (equal); formal analysis (equal); investigation (equal); methodology (equal); project administration (equal); visualization (equal); writing – original draft (equal); writing – review and editing (equal). **Shanqian Li:** Data curation (equal); formal analysis (equal); investigation (equal); visualization (equal); writing – review and editing (equal). **Yue Hu:** Data curation (equal); formal analysis (equal); investigation (equal); visualization (equal); writing – review and editing (equal). **Zongyi Zhang:** Data curation (equal); formal analysis (equal); investigation (equal); visualization (equal); writing – review and editing (equal). **Guixian Wu:** Data curation (equal); formal analysis (equal); investigation (equal); visualization (equal); writing – review and editing (equal). **Yuling Liu:** Data curation (equal); formal analysis (equal); investigation (equal); visualization (equal); writing – review and editing (equal). **Lin Yang:** Funding acquisition (equal); resources (supporting). **Yue Huang:** Funding acquisition (equal); resources (equal). **Wenjun Lu:** Funding acquisition (equal); resources (equal). **Lina Hu:** Conceptualization (equal); funding acquisition (equal); methodology (equal); project administration (equal); resources (equal); supervision (equal); writing – review and editing (equal).

## FUNDING INFORMATION

This work was supported, in part, by the National Natural Science Foundation of China (NSFC, China) (Nos. 82,060,099, 81,760,091 and 81,800,380), Guangxi Science and Technology Base and Special Talents (No. 19,245,054) and the First Affiliated Hospital of Guangxi Medical University introduced high‐level talents scientific research fund (No. 2,022,004).

## CONFLICT OF INTEREST STATEMENT

No potential conflict of interest was reported by the author(s).

## Data Availability

The authors will supply the relevant data in response to reasonable requests.
